# Supplementary material for: Patterns of Intron Gain and Loss in Fungi
Source: PLoS Biol. 2004 Nov 30;2(12):e422. doi: 10.1371/journal.pbio.0020422 (PMC532390; doi:10.1371/journal.pbio.0020422)
Supplement: Table S1 — Also available at http://genes.mit.edu/NielsenEtAl/. (4.3 MB ZIP). [file pbio.0020422.st001.zip › NielsenEtAl/html/583.html]

AN6013.1.NCU04381.1.MG01552.1.FG09590.1


```
 CLUSTAL W (1.82) Multiple Sequence Alignments - Introns Inserted


Sequence 1: MG01552.1	283 aa
Sequence 2: FG09590.1	305 aa
Sequence 3: NCU04381.1	309 aa
Sequence 4: AN6013.1	327 aa
Alignment Length: 363 aa
Number Identitical Residues: 77 aa
Alignment Score (without introns) 4794


MG01552.1 	-------------------------MEDPEEE1TFILFSQDIPSQNLGFIDPSASTIELT
NCU04381.1	MERHHEGGCEELESFLRLLGKEIKDLDDADEE1TYDLYAQDLPSSDLGIIDPKTTKLQLT
FG09590.1 	-----------MEFINDLLPNLEPEVQDAEEE1TFYLYAQPIPSMNLGFIDARATSVDVS
AN6013.1  	-----------MGDLISFLSTIGQEVEDADEE1SFLLFSQDIPSSNLGFVDSKASTVQIT
          	                    .   .::*.:** :: *::* :** :**::*. ::.::::

MG01552.1 	VAGRDLTIHQSPAILSSTRSGGTTGAV1VWKVTPLFAEWISTPDN-ILASAGVISRSSSV
NCU04381.1	VAGRELTIHQSPGILSSARAGGTTG--~------------------------------AV
FG09590.1 	VGDRDFTIHQSPTVLSSTRSGGTTGAV1IWKIAPTFATWLSSPSNPILTKINLAN--ASI
AN6013.1  	VHEREYTVHQSPTLLSSSRAGGTTGAV1LWKITPLFASWVSNTTNPFWTDNVLTS-ASTI
          	*  *: *:**** :***:*:*****:    . :.  :   :.. ..  :.    . ::::

MG01552.1 	~LELGCGTSAVVGLVLAPSVA-------SFTMTDQSYVARLVGQNMQENNNSSVKSKAS-
NCU04381.1	1IELGAGTSAIVGLLLAPRVG-------RYVLTDQAYVSKLVERNIHENWSSVVQQQAPF
FG09590.1 	~LELGCGISPLSALALGPRVS-------RYVLTDQSYVQRLLQKNIDENFSSAFSSGTST
AN6013.1  	~VELGCGISALTALALSPLLEPESNGDGHYIATDQEYVHRLFRQNLQSNPPKPQRTQASS
          	 :***.* *.: .* *.* : ..:.... :  *** ** :*. :*:..*  .     :. 

MG01552.1 	----------KGRRA---------------KSVPQQQQQ-QQALRFMPLDWETDQVTT--
NCU04381.1	VGSGGGGQGGKGKKGGGGGGGGKGSKKASTSSTTGEEGP-GERLVFRALDWEQDTPTS--
FG09590.1 	P------TGGRGRKK---------------RNAHGPIAP-QSNIRFTTLDWETDEVTP--
AN6013.1  	N------QGRKGKRG---------QKASASSSIQGDTAHSHSNITFTALDWETDHPETLK
          	        . :*::          .. ::: .       : . : * .**** *   . .

MG01552.1 	-------SLIGSPHAKSFDLVIACDCIYNESLIEPLAQTCVDACRLREL-EAAPSSDGAS
NCU04381.1	-------SLA-QPVAASFDLVVACDCIYNEALVDPFVTTCVDICKLRGS-SST-SDNGGR
FG09590.1 	-------SLVGSGDARSFDAVVACDCVYNYALVDPFVQTCADACRLRLS-DSALSDNDER
AN6013.1  	AQVVREGGQQEETDDPGFDLLLSCDCIYNEALIAPYVRTCAEIARLRPSPSTSTGIGTAR
          	:.   ...   .    .** :::***:** :*: * . **.: .:**  ..:: . .   

MG01552.1 	LPTVCVVAQQLRDPVIFESWIRRFHRSFQVWRVPDANLPEGLRS------TSGFVVHVGF
NCU04381.1	-PTVVVVAQVLRTSDVFEAWIKRFVQDFRCWRVPDDLMIDGLKSEGGGGGTSGFVVHVGV
FG09590.1 	RPCICVIGQQLRSDEVFESWLKAFSASFHVWRVSDNALPEELRS------TAGFVVHVGI
AN6013.1  	KPTVCVVAQQQRSPDVFEAWLRETMRYFRVYRLNDDVLGESLGVG------SGYLVHGLV
          	 * : *:.*  *   :**:*::     *: :*: *  : : *         :*::**  .

MG01552.1 	LR----
NCU04381.1	LREGN-
FG09590.1 	LREELK
AN6013.1  	LKD---
          	*:.
```
